# Precocious pseudopuberty due to an autonomous ovarian follicular cyst: case report with a review of literatures

**DOI:** 10.1186/1756-0500-6-319

**Published:** 2013-08-12

**Authors:** Hee Suk Chae, Chul Hee Rheu

**Affiliations:** 1 Department of Obstetrics and Gynecology, Research Institute of Clinical Medicine, Chonbuk National University Medical School, Jeonju, South Korea

**Keywords:** Precocious pseudopuberty, Autonomous ovarian follicular cyst, Laparoscopic

## Abstract

**Background:**

Small follicular cysts are commonly found in the ovaries of prepubertal girls, and in most cases, they are of no clinical importance. These cysts are usually self-limiting and resolve spontaneously. However, occasionally, these cysts may enlarge and continue to produce estrogen, resulting in signs of sexual precocity. Here, we report a case of precocious pseudopuberty associated with an autonomous ovarian follicular cyst.

**Case presentation:**

A 5.9-year-old girl initially presented to a local clinic with vaginal bleeding and a large unilateral ovarian cyst. At 6 months after the initial acute episode, the patient visited our hospital as the ovarian cyst had persisted and increased in size. Endocrinological examination showed elevated estrogen levels and suppressed gonadotropin levels on GnRH stimulation test. Also, no skin pigmentation or bone anomaly was noted. Based on these observations, laparoscopic cystectomy was performed, and histologic analysis confirmed the diagnosis of a follicular cyst. After the laparoscopic cystectomy, the patient’s hormone levels returned to normal and no ovarian cyst was detected by ultrasound.

**Conclusions:**

As autonomous ovarian cysts are usually self-limiting disorder, no treatment is necessary. Therefore, surgical management should be deferred as long as possible to avoid the risk of repeat surgery, as pseudoprecocious puberty due to autonomous ovarian cysts can resolve spontaneoulsy and frequently recurs. Precocious pseudopuberty with an ovarian cyst may be due to granulosa cell tumor or may be one symptom of the McCune-Albright Syndrome (MAS). A careful longer-term follow up of patients with autonomous ovarian cysts and/or molecular studies may be necessary in such cases.

## Background

Precocious puberty (PP) is defined as the development of secondary sexual characteristics before the age of 8 years in girls. Precocious puberty has also been further subclassified as gonadotropin-dependent, deemed central precocious puberty or true precocious puberty, or gonadotropin-independent, called pseudo-precocious puberty or peripheral precocious puberty. The latter generally results from excess sex steroids in the absence of activation of the hypothalamic-pituitary-gonadal axis, and may be associated with recurrent ovarian cysts, chronic primary hypothyroidism, adrenal and gonadal tumors, and McCune- Albright syndrome (MAS) [1]. Autonomous functional ovarian follicular cysts in prepubertal girls are rare, but the most common cause of gonadotropin-independent precocious puberty. Millar et al. reported that ovarian cysts are prevalent in 2% to 5% of prepubertal girls, and 5% of ovarian cysts in young girls are found to be autonomous ovarian cysts [2,3]. Here, we report the case of a 5.9-year-old girl who presented with signs of isosexual precocity due to a large ovarian cyst.

## Case presentation

A 5.9-year-old girl visited a local gynecologist because of sexual precocity with signs of vaginal bleeding and breast development. There, the patient’s basal estradiol level was obtained and a pelvic ultrasonography was performed. The basal estradiol level was 66 pg/ml, and the initial ultrasonographic study of the patient’s abdomen and pelvis revealed a 3.7 cm cystic tumor of the right ovary. Until the age of 6.5 years, she continued to experience repeated episodes of vaginal bleeding, at which time she visited our hospital for further evaluation and management. Her past and family medical history was unremarkable except for a past history of hydrocephalus with ventriculoperitoneal shunting (V-P shunt) the age of 4 months old. She exhibited no neurological signs or symptoms.

On physical examination, she had enlarged breasts (Tanner stage 2–3) with pigmentation of the areola. She was absent of axillary and pubic hair. Her external genitalia and clitoris were normal for her puberty stage. Her height and weight measured in the 50–75 percentile and 25 percentile, respectively. No signs of McCune-Albright syndrome (MAS), including café-au-lait skin pigmentation and bone deformity were identified on physical examination.

**Figure 1:**
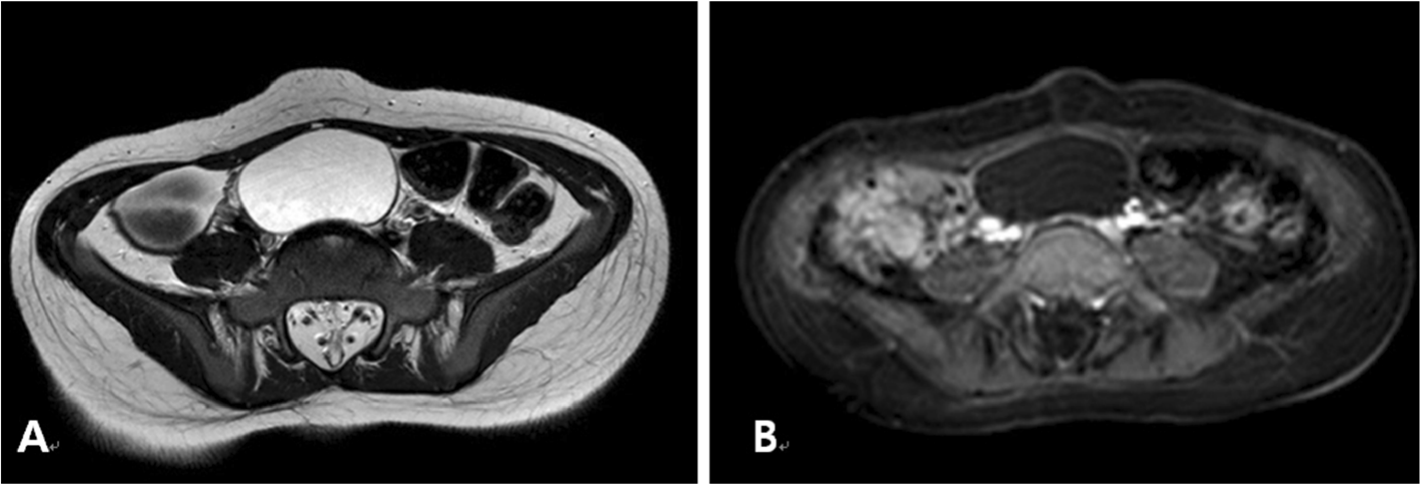
An Axial T2-weighted MR image (A) and T1-weighted contrast enhanced MR image (B) show an ovoid to round cystic mass with well-defined margins and a thin wall without any internal component.

A pelvic ultrasound revealed an echo-free right ovarian cyst with a smooth lining and no septum or solid area within the cyst cavity, measuring 5.09 × 4.16 cm. The uterus was enlarged with a length of 5.45 cm (normal ≤ 3 cm) and had a prominent endometrium. Magnetic resonance imaging (MRI) confirmed the presence of a unilocular right ovarian cyst measuring 5.0 × 5.4 cm without a solid component (Figure 1). No other abdominal or pelvic abnormalities were noted. The patient’s bone age was appropriate for her chronological age. Subsequent testing of her brain MRI showed callosal dysgenesis and a ventriculoperitoneal shunt, but was otherwise normal.

Hormonal analysis revealed an elevated level of estradiol (19.8 pg/ml, normal < 10 pg/ml) and suppressed baseline FSH and LH levels (FSH, 0.3 mIU/ml (normal > 4); LH, 0.07 mIU/ml (normal >0.3)). Serum gonadotropin responses to gonadotropin releasing hormone stimulation were prepubertal. Thyroid-stimulating hormone, 17-hydroxy progesterone, adrenocorticotropic hormone, cortisol and prolactin were all in their normal range. Molecular analysis of genomic DNA obtained from peripheral lymphocytes in our patient did not reveal the presence of a GNAS 1 gene mutation.

**Figure 2:**
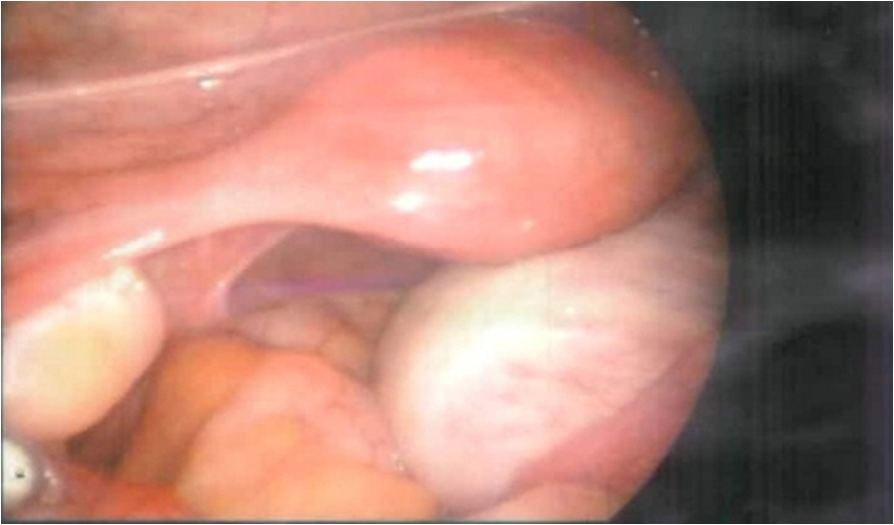
Laparoscopic finding shows an enlarged right ovary.

**Figure 3:**
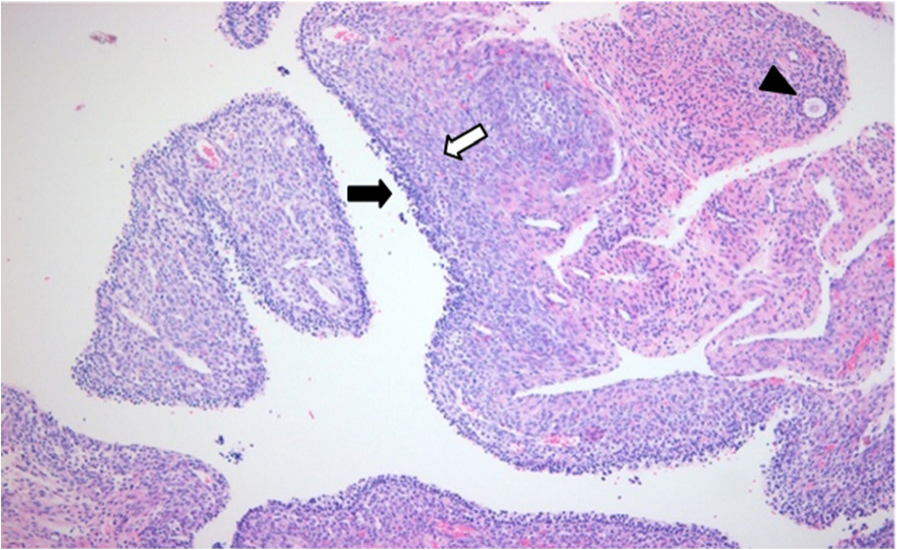
**Histopathologically, a follicular cyst wall is lined by an inner granulosa layer (black arrow) with an outer theca interna cell layer (white arrow).** Surrounding ovarian stroma composed of whorls of plump fibroblastic spindle cells. Primordial follicles are found scattered irregularly in clusters (arrow head).

Based on these observations, laparoscopic cystectomy was performed (Figure 2). Evaluation of a frozen section revealed a benign follicular cyst. Histopathologic examination further exposed a follicular cyst wall lined by an inner granulosa layer with an outer theca interna cell layer and surrounding ovarian stroma composed of whorls of plump fibroblastic spindle cells. Also, primordial follicles were found scattered irregularly in clusters (Figure 3).

Her postoperative course was uneventful, and she was discharged on postoperative day 4. At one month follow-up, breast development had regressed and estradiol concentration had returned to a prepubertal range of 0.01 pg/ml. Seven months later, she remained well without relapse and ultrasound showed normal prepubertal ovaries.

## Discussion

Polycystic ovaries are commonly found in normal girls of all ages, and the majority of cysts in the prepubertal children are of no clinical importance [4]. In one series from Jordan, 89.2% of 65 ovarian cysts in girls ranging in age from 2 to 9 years resolved spontaneously within 6 months, from which the authors concluded that the majority of cysts are not clinically significant and resolve within 6 months [5]. However, occasionally, these cysts may enlarge and continue to produce estrogen, resulting in signs of sexual precocity, including breast development, vaginal discharge or bleeding, and swelling of the labia minora [4]. Therefore, all prepubertal girls with ovarian cysts should undergo careful physical examination to rule out the signs of increased hormone production associated with central precocious or pseudoprecocious puberty [6].

The etiologic diagnosis of early sexual precocity is based on careful history and physical examination, measurement of bone age, estradiol levels, GnRH-stimulation test, and pelvic ultrasound examination [3]. Currently, GnRH-stimulation test is considered the gold standard for correctly diagnosing children with precocious puberty [7]. Gonadotropin-dependent and gonadotropin-independent precocious puberty can be distinguished by differences in the responses to GnRH-stimulation testing, as GnRH-stimulated luteinising hormone (LH) and follicle stimulating hormone (FSH) concentrations are suppressed in pseudoprecocious puberty, but increased in central precocious puberty [8]. Ultrasonography is also a valuable diagnostic tool for evaluating sexual precocity caused by primary ovarian cysts [9]. In the patient presented here, GnRH-stimulation test results were compatible with pseudoprecocious puberty and pelvic ultrasound examination revealed a unilateral large ovarian cyst. Magnetic resonance study showed no adrenal mass. The source of sex hormones was then localized to autonomous estradiol production from ovarian cyst. With these findings, diagnosis of the autonomous functional ovarian follicular cyst was able to be made.

Autonomous functional ovarian follicular cysts are the most common cause of gonadotropin-independent precocious puberty in girls [10]. Typically, serum estrogen levels are elevated, but not always (due to regression of the cyst), and both basal and GnRH-stimulated gonadotropin concentrations are low [10]. Ovarian ultrasonography often reveals one or more unilateral or bilateral ovarian cyst. Millar et al. reported that observations of small, unilocular ovarian cysts of less than 1 cm in diameter in prepubertal girls are clinically insignificant, whereas ovarian cysts associated with precocious pseudopuberty are generally larger than 2 cm in diameter [2]. Accordingly, Fakhry et al. reported the presence of autonomous ovarian cysts (2.2 to 5.5 cm) in three girls who initially presented with sexual precocity [9]. Reportedly, 20%~30% of ovarian enlargements in young girls are caused by follicular cysts, but only a few are associated with sexual precocity, as even large sized cysts are not hormonally active [11]. Ovarian tumors, including granulosa cell tumors, are the least common cause of precocious puberty, but should be differentiated from autonomous ovarian cysts, owing to their poor prognosis [3,12]. Furthermore, autonomous ovarian cysts may be one symptom of McCune-Albright syndrome (MAS), arising before emergence of the characteristic skin pigmentation or skeletal lesions. Rodriguez-Macias et al [13],. who examined the clinical outcomes of seven girls who presented with pseudoprecocious puberty caused by isolated autonomous ovarian cysts, reported that clinical evidence of MAS ultimately appeared in three girls of the seven girls. MAS is a non-inherited disorder caused by a somatic mutation of the alpha subunit of the G protein (encoded by the GNAS1 gene) [10]. Accordingly, molecular analysis of genomic DNA can be performed to uncover the presence of this somatic mutation for early detection of MAS, which was undetected in our patient.

Autonomous ovarian cysts are self-limited in most and typically require no treatment [3,10,13]. Surgical intervention is indicated only in the rare case of ovarian torsion, or if development of secondary sexual characteristics and if the ovarian cyst fails to resolve or decrease in size within 3 months [3]. In the presented patient, laparoscopic cystectomy was performed because sexual precocity remained unchanged and the cyst significantly increased in size 6 months after her initial visit. Pseudoprecocious puberty due to autonomous ovarian cyst is often transient and frequently recurrent. However, relapses of autonomous ovarian cysts resulting in prolonged or repeated estrogen exposure can precipitate early maturation of the hypothalamic-pituitary-gonadal axis, resulting in progression from precocious pseudopuberty to central precocious puberty, called “combined precocious puberty,” which may require GnRH therapy [3]. Engiz et al. reported that aromatase inhibitors can be used to effectively treat pseudoprecocious puberty due to recurrent autonomous ovarian cysts [14]. However, there is no consensus for the management of recurrent autonomous ovarian cysts in prepubertal girls, and long-term studies are needed.

## Conclusions

Surgical management should be deferred as long as possible to avoid the risk of repeat surgery, as pseudoprecocious puberty due to autonomous ovarian cysts can resolve spontaneously and frequently recurs. However, if an attempt at surgical intervention is made, conservation of normal ovarian tissue for future fertility should be considered. As recurrent autonomous ovarian cysts can also be an early manifestation of McCune-Albright syndrome, a careful longer-term follow up of patients with recurrent ovarian cysts and/or molecular studies may be necessary in such cases [3].

### Consent

Written informed consent was obtained from the patient’s legal guardian for publication of this case report and any accompanying images. A copy of the written consent is available for review by the Editor of this journal.

## Abbreviations

GnRH stimulation test: Gonadotropin releasing hormone stimulation test; MAS: McCune-Albright syndrome; GNAS: Guanine nucleotide binding, alpha stimulating complex.

## Competing interests

The authors declare that they have no competing interests.

## Authors’ contributions

HSC and CHR conceived of the study, and participated in its design and coordination and drafted the manuscript. Both authors read and approved the final manuscript.

## Authors’ information

CHR is a MD/PhD and a director of Dept. of Ob. & Gyn. of Chonbuk National University Medical School in South Korea. HSC is a MD/ clinical Professor and has a position in the Chonbuk National University Medical School in South Korea.
